# Bone marrow infiltration pattern in patients with intravascular large B‐cell lymphoma diagnosed by random skin biopsy

**DOI:** 10.1002/jha2.66

**Published:** 2020-07-20

**Authors:** Kosei Matsue, Yoshiaki Abe, Kentaro Narita, Hiroki Kobayashi, Akihiro Kitadate, Daisuke Miura, Masami Takeuchi, Kengo Takeuchi

**Affiliations:** ^1^ Division of Hematology/Oncology Department of Internal Medicine Kameda Medical Center Kamogawa Japan; ^2^ Division of Pathology The Cancer Institute Japanese Foundation for Cancer Research Tokyo Japan

**Keywords:** bone marrow biopsy, incisional random skin biopsy, intravascular large B‐cell lymphoma

## Abstract

We retrospectively analyzed bone marrow (BM) infiltration pattern in consecutive 30 intravascular large B‐cell lymphoma (IVLBCL) patients diagnosed by random skin biopsy (RSB). BM infiltration of lymphoma was observed in 18 patients (60.0%), including five patients with the intrasinusoidal pattern with minimal extravasation, eight patients with the mixed of intrasinusoidal and scattered/interstitial or nodular infiltration, and five patients with the nodular/diffuse pattern. Twelve patients were negative for lymphoma infiltration. BM histology of patients with IVLBCL were diverse and frequently discordant with those of other site of IVLBCL lesions. BM biopsy had a poorer diagnostic performance for detecting intravascular features.

## 1 INTRODUCTION

Intravascular large B‐cell lymphoma (IVLBCL), an extremely rare form of extranodal large B‐cell lymphoma, is characterized by selective proliferation of lymphoma cells within the lumina of small blood vessels [1]. IVLBCL diagnosis remains challenging because it usually lacks approachable nodal or tumorous lesions and targeted organ biopsies are often impracticable. Bone marrow biopsy (BMB) has been widely used as a less‐invasive diagnostic method for patients suspected IVLBCL in many Western and Asian countries [2]; however, its diagnostic sensitivity for IVLBCL remains unclear. Our recent study indicates that the incisional random skin biopsy (RSB) has an excellent sensitivity for IVLBCL [3]. However, we noted that some patients diagnosed as IVLBCL using RSB showed a different pathognomonic lymphoma infiltration patterns on BM histology [4]. This prompted us to reconsider the diagnostic value of BMB in patients suspected of IVLBCL. The objectives of this study were to investigate the correlations between the histopathology of BMB and incisional RSB as well as to determine the diagnostic value of BMB for IVLBCL.

## 2 MATERIALS AND METHODS

### 2.1 Study design and patients

We retrospectively analyzed 30 consecutive patients diagnosed as IVLBCL using incisional RSB at Kameda Medical Center, Japan between June 2006 and December 2019. Patients diagnosed as IVLBCL other than skin or bone marrow and were excluded from the study. Incisional RSB were performed before initiating any form of therapy who were suspicious of IVLBCL [3]. Bone marrow specimens were obtained from the iliac crest as a part of initial screening for extend of disease. RSB were obtained from three separate, fat‐containing areas as previously described [3, 4, 5]. Two patients were diagnosed as having IVLBCL by lung and adrenal biopsy, respectively and these two patients were not received RSB. Histopathological findings were interpreted by an expert hematopathologist (K.T.). Written informed consent was obtained from all participating patients or their families. The study was conducted according to the Declaration of Helsinki and was approved by the review board of Kameda Medical Center.

### 2.2 Identification and categorization of marrow involvement in patients with IVLBCL

**FIGURE 1:**
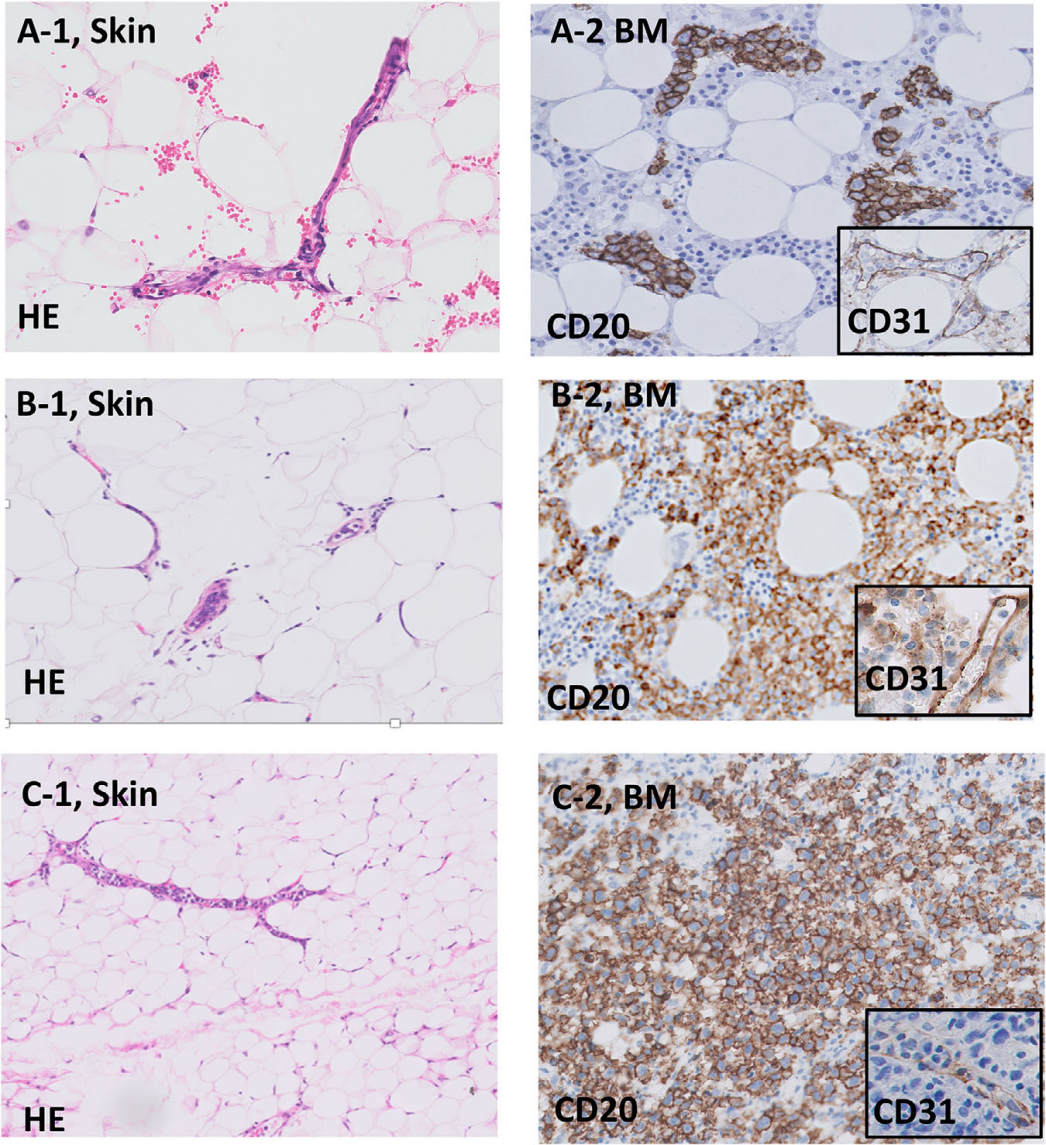
Bone marrow histology categories and comparison of histological findings between incisional random skin biopsy findings and bone marrow biopsy in patients with intravascular large B‐cell lymphoma. A, The intra‐sinusoidal pattern with minimum extravasation. CD31‐immunostaining was used to identify endothelial cells. Arrows indicate the localization of lymphoma cells within the sinusoids. B, Representative intra‐sinusoidal infiltration with the scattered/interstitial extravasation pattern in a patient with IVLBCL diagnosed using random skin biopsy. Although, neoplastic tumor cells were confined into capillary vessels in skin biopsy, lymphoma infiltration was also observed scattered interstitially extra‐sinusoidal space as well as intra‐sinusoidal space in the bone marrow. C, Bone marrow histology in a patient with the nodular/diffuse pattern diagnosed with IVLBCL using RSB. CD20 positive lymphoma cells proliferate diffusely in the bone marrow

The presence of lymphoma was identified based on immunohistochemistry using an antibody specific for CD20 or CD79a. The anti‐CD31 antibody was used to demonstrate the endothelial cells for confirming the localization of lymphoma cells within the capillary lumina. The marrow infiltration pattern of IVLBCL was categorized as a negative pattern, intrasinusoidal pattern with or without minimal extravasation [6] (Figure 1A), intrasinusoidal pattern with substantial scattered/interstitial extravasation (Figure 1B), or nodular/diffuse pattern (Figure 1C) according to our recent report [4]. Patients who had an intrasinusoidal pattern with substantial scattered/interstitial extravasation or nodular/diffuse pattern alone without definite intravascular lesions in tissues other than bone marrow were not considered as IVLBCL.

### 2.3 Statistical analysis

Relationships between baseline characteristics and marrow infiltration patterns were assessed using one‐way analysis of variance, the Kruskal‐Wallis test, or chi‐squared tests as appropriate. The probabilities of overall survival (OS) were estimated using the Kaplan‐Meier method and compared using the log‐rank test. A two‐tailed *P*‐value of <.05 was considered statistically significant. Statistical analysis was performed using the R software, version 3.1.2 (R Foundation for Statistical Computing, Vienna, Austria).

## 3 RESULTS

### 3.1 Demographic and baseline patient characteristics of patients

Baseline characteristics of all the IVLBCL patients are shown in Table 1. All 30 patients showed typical features of IVLBCL on incisional RSB histology. The median patient age was 72 years and 16 patients (57.1%) were male. The median observation period was 21.5 months. Twenty‐five patients (89.3%) presented with cytopenia (neutrophils < 1.5 × 10^9^/L, hemoglobin < 10.0 g/dL, or platelets < 100 × 10^9^/L) and 13 (46.4%) had hemophagocytic syndrome. All but two patients underwent immunochemotherapy with rituximab plus CHOP (cyclophosphamide, adriamycin, vincristine, and prednisolone) (n = 25) or CHOP‐like (n = 3) regimens. Two patients did not received chemotherapy because of refusal and died before diagnosis.

**TABLE 1:** Baseline clinical characteristics of patients

		Bone marrow infiltration pattern				
	All patients	Negative (%)	Intrasinusoidal with minimal extravasation (%)	Intrasinusoidal with Scattered/interstitial (%)	Nodular/diffuse (%)	
Clinical factors	n = 30	n = 12 (40)	n = 5 (16.7)	n = 8 (26.7)	n = 5 (16.7)	*P*‐value
Age, years [median (range)]	71 (54, 87)	72 (60, 83)	81 (63, 87)	69 (57, 74)	76 (54, 83)	.16
Sex, Male (%)	18 (60.0)	5 (41.7)	3 (60.0)	6 (20.0)	4 (40.0)	.26
WBC, ×10^9^/L [median (IQR)]	5500 (3500, 8200)	8200 (5525, 8425)	3500 (3000, 4500	5200 (3450, 6200)	5700 (5500, 6000)	.339
Hemoglobin, g/dL [median (IQR)]	8.9 (8.0, 12.0)	9.3 (8.0, 11.3)	7.6 (7.0, 9.0)	11.0 (10.0, 12.0)	10.0 (9.0, 12.0)	.125
Platelet, ×10^9^/L [median (IQR)]	7.4 (5.0, 15.0)	8.3 (7.0, 14.0)	6.5 (6.0, 19.0)	5.9 (3.9, 12.3)	4.0 (3.0, 6.0)	.251
Hemophagocytic syndrome (%)	12 (40.0)	6 (50.0)	1 (20.0)	4 (50.0)	1 (20.0)	.055
Splenomegaly (%)	23 (82.1)	11 (91.7)	2 (40.0)	7 (87.5)	3 (60.0)	.71
LDH, U/L [median (IQR)]	896 (589, 1285)	773 (582, 1172)	427 (416, 1120)	1248 (987, 1329)	847 (713,1112)	.489
sIL‐2R, U/mL [median (IQR)]	6780 (4695, 10296)	6347 (4119, 9287)	9593 (2679, 10060)	6985 (4743, 11717)	7120 (6780, 19558)	.583
Treatment (%)						
Rituximab plus CHOP	25 (89.3)	12 (92.3)	4 (80.0)	5 (80.0)	5 (100)	NA
Rituximab plus EPOCH	3 (10.0)	1 (7.7)	1 (20.0)	1 (20.0)	0 (0.0)	
Other	2 (6.7)	0 (0.0)	0 (0.0)	1	0 (0.0)	NA
Outcome (%)						
Alive	16 (53.3)	6 (50.0)	3 (60.0)	5 (62.5)	2 (40.0)	NA
Dead	14 (47.7)	6 (50.0)	2 (40.0)	3 (37.5)	3 (60.0)	

Abbreviations: CHOP, cyclophosphamide, adriamycin, vincristine, and prednisone; EPOCH, etoposide, prednisolone, vincristine, cyclophosphamide, and adriamycin; IQR, interquartile range; LDH, lactate dehydrogenase; sIL‐2R, soluble interleukin‐2 receptor; IQR, inter quarter range; NA, not assessed.

### 3.2 Diagnostic performance of BMB in IVLBCL patients initially diagnosed using RSB

Marrow involvement of large B‐cell lymphoma was detected in 18 patients (60.0%) but not in the remaining 12 (40%) patients. Accordingly, the positivity of BMB for large B‐cell lymphoma diagnosis, not IVLBCL, was 53.6%. An intrasinusoidal marrow infiltration pattern with or without minimal extravasation that was diagnostic for IVLBCL was observed only in five patients. Therefore, the sensitivity of BMB for IVLBCL diagnosis was only 16.7% (95% confidence interval, 5.6‐34.7%).

### 3.3 Prevalence and clinical relevance of each marrow histology pattern

The intrasinusoidal pattern with or without minimal extravasation, the intrasinusoidal pattern with substantial scattered/interstitial infiltration, and the nodular/diffuse patterns were observed in five, eight, and five patients among 18 patients with positive BMB, respectively. We found four patients had mixed marrow patterns in a single marrow specimen: one patient with the intrasinusoidal and scattered interstitial patterns and three with the scattered interstitial and nodular/diffuse patterns. Clinical characteristics of patients according the marrow patterns are summarized in Table 1. Patients with a nodular/diffuse pattern had appeared to have higher lactate dehydrogenase (LDH) levels. No inter‐group differences in other clinical factors, including treatment approaches, were detected. In addition, no survival difference was observed between the patients with or without bone marrow infiltration (median, not reached vs 27.7 months; Figure S1).

## 4 DISCUSSION

In the present study, we found considerable discordance between BMB‐ and RSB‐based histological findings in IVLBCL patients. To the best of our knowledge, no other study has compared the diagnostic performances and histological findings between BMB and RSB in IVLBCL patients using a sizeable cohort.

BMB is widely accepted as a screening test for IVLBCL, especially in patients without apparent skin lesions [2]. However, in our study, BMB exhibited approximately 60% sensitivity for detecting lymphoma involvement and only 16.7% specificity for identifying the IVLBCL lesions, respectively. The low sensitivity of BMB for IVLBCL diagnosis is of particular importance because this disease has several clinical and prognostic peculiarities (eg, neural or pulmonary involvement) that distinguish it from other large B‐cell lymphomas [7, 8, 9, 10, 11, 12]. Our findings suggest that a significant proportion of IVLBCL patients might have been missed and/or diagnosed with diffuse large B‐cell lymphoma (DLBCL) of the bone marrow if they were not evaluated using incisional RSB [13].

In this study, we assessed marrow histology in patients diagnosed with IVLBCL using the IVLBCL lesions other than bone marrow. The high incidence of negative marrow infiltration (13/28, 46.4%) in RSB positive IVLBCL patients may support the notion that RSB may detect IVLBCL at an early disease stage. Indeed, patients with scattered/interstitial or nodular/diffuse marrow patterns tended to have higher LDH levels than those without such patterns; furthermore, patients with negative marrow infiltration experienced relatively longer OS rates despite undergoing similar treatments, although this difference was likely not statistically significant probably because of the small sample size.

Intriguingly, the BM histological findings in IVLBCL patients were highly heterogeneous. Approximately 20% of the patients showed unequivocal nodular/diffuse infiltration that are incongruous with both skin histology and the expected disease‐related features [2]. The findings appear to be consistent with those in several previous studies, in which patients with rare IVLBCLs presented with concomitant bulky cardiac DLBCL masses, [14] nodal DLBCL lesions at relapse, [15] or extensive extravascular proliferation and tumour growth [16]. It is possible that IVLBCL may merely be an angiotrophic manifestation of a certain type of DLBCL, and may change its histological features according to the involved sites and microenvironments. These phenomena might also be associated with the loss of adhesion molecules in IVLBCL [17]. Alternatively, IVLBCL could be included in the recent proposed category of distinct subtype of “bone marrow‐liver‐spleen” (BLS) type DLBCL that is characterized by bone marrow involvement of large B‐cell lymphoma with or without liver and/or spleen, but no lymph node or other extranodal sites, and is often associated with fever, anemia, and hemophagocytosis [18].

This study has several limitations, including its retrospective nature and single‐institution design. Bone marrow histology was usually performed unilateral iliac crest, while RSB was performed from three different sites of the body. However, this study included the largest number of IVLBCL patients investigated to date, and they were thoroughly evaluated using both pretreatment BMB and incisional RSB.

In conclusion, this study demonstrated the heterogeneity of marrow histology and the low incidence of pathognomonic, intra‐sinusoidal marrow infiltration patterns in newly diagnosed IVLBCL patients diagnosed by RSB. BMB was found to have a low sensitivity for detecting intravascular features. These findings may provide new insights into IVLBCL biology and shed light on the development of useful diagnostic strategies for IVLBCL.

## AUTHOR CONTRIBUTIONS

K.M. and Y.A. planned and designed the study, collected data, wrote the manuscript, and provided patient care. K.N., H.K., A.K., D.M., and M.T. provided patient care. K.T. interpreted all histology findings. All authors reviewed and approved the manuscript.

## CONFLICT OF INTEREST

K.M. received honoraria from Celgene Corporation, Janssen Japan, and Takeda pharmaceutical company. For the remaining authors, no conflict of interest was declared.

## Supporting information

SUPPORTING INFORMATION
