# Supplementary material for: Bone marrow infiltration pattern in patients with intravascular large B‐cell lymphoma diagnosed by random skin biopsy
Source: EJHaem. 2020 Jul 20;1(1):281–5. doi: 10.1002/jha2.66 (PMC9175664; doi:10.1002/jha2.66)

Supplement Figure 1,

Overall survival of Patients with IVLBCL according to the presence or absence of bone marrow infiltration

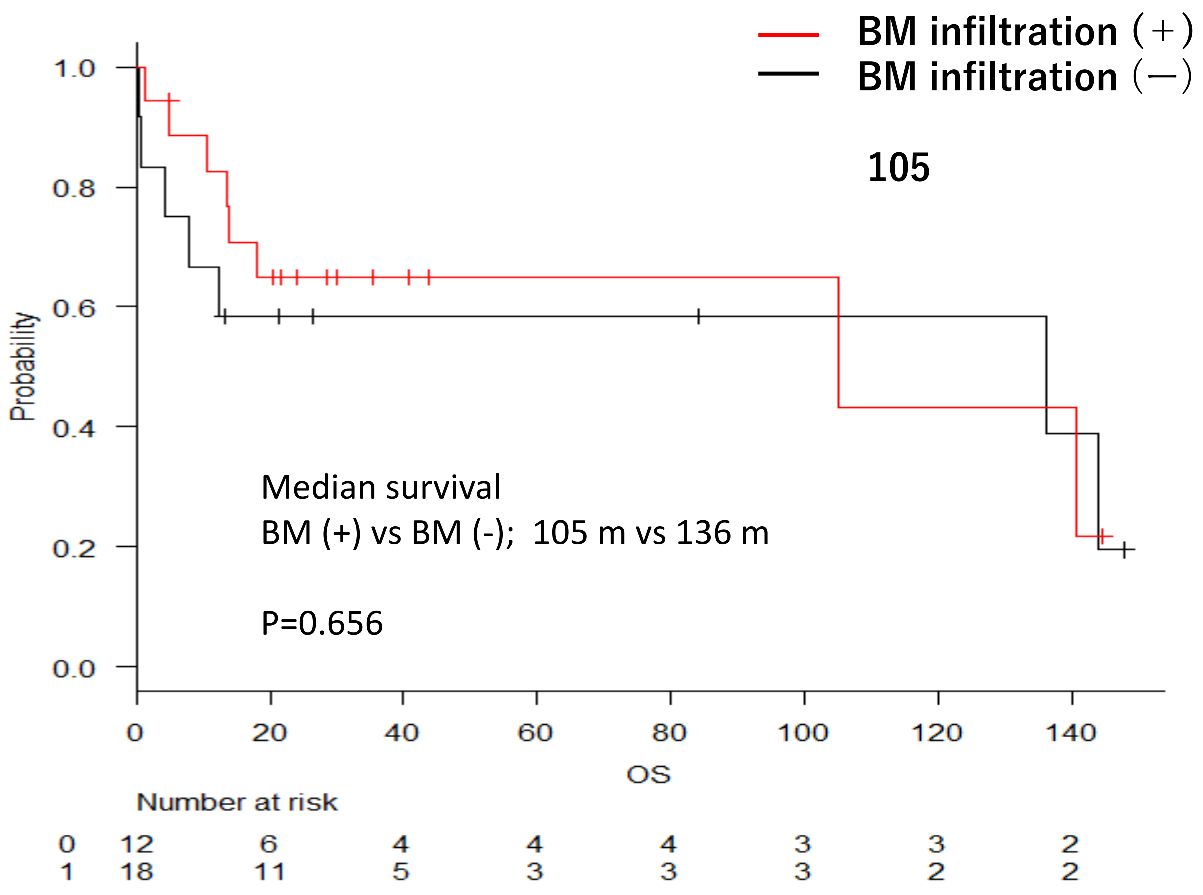

Supplement: Supplementary file 1 — SUPPORTING INFORMATION [file JHA2-1-281-s001.pdf]
